# Association between daily ambient temperature and drug overdose in Tokyo: a time-series study

**DOI:** 10.1265/ehpm.21-00044

**Published:** 2022-09-28

**Authors:** Ananya Roy, Md Ashraful Alam, Yoonhee Kim, Masahiro Hashizume

**Affiliations:** 1 Department of Global Health Policy, Graduate School of Medicine, The University of Tokyo, Tokyo 113-0033, Japan; 2 Department of Global Environmental Health, Graduate School of Medicine, The University of Tokyo, Tokyo 113-0033, Japan

**Keywords:** Suicide attempt, Self-harm, Drug overdose, Ambient temperature, Weather factors, Time-series study

## Abstract

**Background:**

Previous studies have reported that high ambient temperature is associated with increased risk of suicide; however, the association has not been extensively investigated with drug overdose which is the most common method of unsuccessful suicidal behavior in Japan. Therefore, this study aims to examine the short-term association between daily mean temperature and the incidence of self-harm attempts by drug overdose in Tokyo, Japan.

**Methods:**

We collected the emergency ambulance dispatch data and daily meteorological data in Tokyo from 2010 to 2014. A quasi-Poisson regression model incorporating a distributed lag non-linear function was applied to estimate the non-linear and delayed association between temperature and drug overdose, adjusting for relative humidity, seasonal and long-term trends, and days of the week. Sex, age and location-specific associations of ambient temperature with drug overdose was also estimated.

**Results:**

12,937 drug overdose cases were recorded during the study period, 73.9% of which were female. We observed a non-linear association between temperature and drug overdose, with the highest risk observed at 21 °C. The highest relative risk (RR) was 1.30 (95% Confidence Interval (CI): 1.10–1.67) compared with the risk at the first percentile of daily mean temperature (2.9 °C) over 0–4 days lag period. In subgroup analyses, the RR of a drug overdose at 21 °C was 1.36 (95% CI: 1.02–1.81) for females and 1.07 (95% CI: 0.66–1.75) for males. Also, we observed that the risk was highest among those aged ≥65 years (RR = 2.54; 95% CI: 0.94–6.90), followed by those aged 15–34 years (RR = 1.25; 95% CI: 0.89–1.77) and those aged 35–64 years (RR = 1.15; 95% CI: 0.78–1.68). There was no evidence for the difference in RRs between urban (23 special wards) and sub-urban areas in Tokyo.

**Conclusions:**

An increase in daily mean temperature was associated with increased drug overdose risk. This study indicated the positive non-linear association between temperature and incomplete attempts by drug overdose. The findings of this study may add further evidence of the association of temperature on suicidal behavior and suggests increasing more research and investigation of other modifying factors.

**Supplementary information:**

The online version contains supplementary material available at https://doi.org/10.1265/ehpm.21-00044.

## Background

Suicide is a complex global phenomenon and one of the most common preventable causes of death worldwide [1]. World Health Organization reports that more than 700,000 people committed suicide in recent days, and for every suicide there are more prior self-harm or suicide attempts [1]. So, A previous self-harm attempt is the most critical risk factor for future suicide attempts and suicide in the general population [1]. According to the Global Burden of Disease Study, in 2016, 817,000 deaths occurred from suicide globally; these results account for 1.49% of all deaths per 100,000 people, making suicide the 18^th^ most frequent cause of death in the world [1, 2].

In Japan, 16 suicides per 100,000 inhabitants occurred in 2019 [3]. Despite a declining trend in suicide in recent years, the rate is still higher than the average among high-income Organization for Economic Co-operation and Development (OECD) nations [3]. One nationwide descriptive study using data on emergency hospital admissions in Japan showed that among patients, the most common method of unsuccessful suicide or self-harm attempts was drug overdose (50%), followed by hanging, jumping from a height, and cutting or piercing other than wrist cutting; [4] while for suicide deaths from 1979 to 2016 was majorly driven by hanging [5]. A study conducted in Akita prefecture have reported male predominance (73.9%) in completed suicides, and contrarily, a female predominance (59.1%) in failed attempts [6]. One European 16 center study hypothesized that, on average completed suicide prevails among males and failed attempts among females [7].

With global climate change, ambient temperature has directly affected human health (e.g., morbidity and mortality) and has become one of the world’s most severe public health concerns [8]. According to a recent study, by 2050, increasing temperatures could increase suicide rates of 1.4% in the United States and 2.3% in Mexico [9]. Likhvar et al. 2011, Page et al. 2007 hypothesized the increased temperature to higher suicide counts is likely to be acting immediately that is increased on the same day of the event [10, 11]. An earlier review study attempted to summarize the associations of climate and weather factors with suicidal behavior [12] and found both positive [13, 14] and negative [15] associations with the temperature, resulting in inconclusive evidence overall. The inconsistent findings of the previous studies might be due to less sophisticated and varied methodologies. However, contemporary studies have indicated evidence for significant positive risk of suicide and suicidal behavior in high temperature [16–22], yet few have found negative relationships [23].

Associations of the season and environmental factors with suicide and suicidal behavior are continued to be a focus of psychiatric research. However, few studies have given attention to the association between temperature and unsuccessful attempts [24–26]. Previous studies found evidence with seasonal components for complete suicide, though less evident in a failed attempt [14, 21]. As far as a subgroup is concerned, in one Turkish study, suicide attempts occurred most frequently during summer in both sexes [25]; while seasonal variations in mean daily attempted suicide figures were seen primarily in women in Great Britain study [27]. A study in Israel revealed that suicide attempt was elevated during summer yet no significant connection for sex subgroup [24].

Given the discrepancy and lack of conclusive reports between suicide attempts or self-harm events and their relationship with temperature, it is worth exploring whether failed attempts are also strongly influenced by short-term ambient temperature variations. These findings would help us to understand better the potential impact of temperature on suicidal behavior in an extensive manner. With increasing threat of global climate change, there might have an impact on mental health related diseases [28]. Any self-harm attempts are assumed to depend on the accessibility and availability of the mean and thus may increase the risk of drug overdose cases as method of attempts. Despite being the most common method, the association of ambient temperature on the drug overdose to our knowledge has not yet been investigated earlier. Therefore, to improve previous findings and to add more knowledge, we aimed to examine the short-term association between temperature and the incidence of self-harm attempts by drug overdose in Tokyo, Japan and examine the potential modification of temperature-drug overdose relationship by sex. age and location groups.

## Methods

### Study area and population

Tokyo, officially Tokyo Metropolis, the capital of Japan, is highly developed, urbanized and the most populated area in the country, with an estimated population of 13,929,280 in an area of 622 km^2^ as of 2019. Tokyo lies in a humid subtropical climate zone (Köppen climate classification Cfa) [29] with hot, humid summer and mildly cool winter with sporadic cold. This study gathered Japanese emergency ambulance dispatch data (EAD) records from the Tokyo Municipal Fire Department from January 2010 to December 2014. All patients diagnosed with drug overdose initially managed by Emergency medical service (EMS) personnel in Tokyo were included in this study. The municipality generally conducts an EMS system, and its response comprises a single-tiered ambulance system dispatched for all patients who need emergency ambulance transportation [30]. Local fire departments operate the EMS system throughout Japan covering entire EAD record services for emergency purposes [31].

### Data collection

The outcome variable in this study was the daily number of drug overdose cases to which the EAD responded that were assumed as self-harm attempts. The emergency patients initially taken to a hospital, the attending physician clinically diagnose the cases with EMS personnel’s help and those patients not taken to the hospitals were managed at the scene. EMS personnel made their diagnosis based on firsthand observations and interview. We obtained daily EAD data from January 1, 2010, to December 31, 2014, from the Japanese Fire and Disaster Management Agency offices across the study region. For each drug overdose case, the dataset included the date and time of the event, primary symptoms, primary diagnosis, and basic information such as age, sex, birthdate, address and nationality. In this study, we initially extracted all ambulance dispatches for suicide attempts and self harm events using various methods from the data. Because there were no definite mentions of *ICD-10* codes [32], we decided to focus on drug overdose cases in the data.

The outcome variable was the number of cases clearly indicating drug overdose and acute drug poisoning. Previous, studies reported the commonly used drugs for self-harm and suicide attempts in Japan, including psychotropic drugs, anti-epileptic drugs, sedatives, hypnotics, and anticonvulsants [33, 34]. We excluded all types of medications used for cardiac diseases, illegal drugs, and all cases of unintentional poisoning or overdose (Table S1). We obtained daily mean temperature and relative humidity data from the Japan Meteorological Agency (JMA). The data were measured at a single weather station in central Tokyo.

### Statistical analysis

We applied Quasi-Poisson distributed lag non-linear models (DLNMs) to fit the time-series data, allowing overdispersion to describe the relationship between the temperature-drug overdose [35]. DLNM allows for the simultaneous estimation of different non-linear functions of the outcome-exposure relation at each lag period and allows for the estimation of cumulative non-linear effects across lags. The methodology is based on the definition of a “cross-basis” function. Here, the cross-basis of DLNM was used to explore and model non-linear and distributed lag structure of temperature over lag 0 to 4 days. We modelled the temperature-drug overdose curve with a natural cubic spline (NCS) with an internal knot placed at 75th percentile (23.5 °C) and lag-response curve was generated with strata function. An NCS function was executed for calendar time (df = 8/year) to control long-term trends and season and relative humidity (df = 3). We controlled the day of the week as an indicator variable. The 1^st^ percentile of mean temperature (2.9 °C) was defined as the reference temperature for calculating relative risks (RRs). Quasi Akaike information criterion (QAIC) was used to evaluate the fitness of models and choose the degree of freedom [36].

The general model is expressed as:

$$\begin{array}{rl} & Y_{t}∼\text{quasi}-\text{Poisson}(\mu_{t})\\ & Log(Y_{t})=\alpha +\beta T_{t,l}+NCS({RH}_{t},\text{df}=3)\\ & \;\;\;+\gamma {DOW}_{t}+NCS(\text{Time},\text{df}=8*5) \end{array}$$

where *Y_t_* denotes the daily count of drug overdose cases on day *t*; *α* is the intercept; *β* is a vector of coefficients for *T_t_*_,_*_l_*; *T_t_*_,_*_l_* signifies a matrix obtained by applying the “cross-basis” DLNM functions to the mean temperature; *l* indicates the lag days, here the lag period is from the previous 4 days till the event day; *NCS*() is the natural cubic spline; RH indicates relative humidity; *DOW*_t_ is an indicator variable signifying the day of the week on day t; *NCS*(*Time*) is a variable for date.

We performed stratified analyses of age group, sex and location. We created the age groups of adolescents and young adults (aged 15–34 years), middle-aged adults (aged 35–64 years), and older adults (aged ≥65 years) The location was divided into urban (23 special wards) and sub-urban (the rest in Tokyo). We used the same reference temperature (2.9 °C) for all drug overdose cases to estimate the RRs for each subgroup.

We also performed a sensitivity analysis to testify the stability of the model by applying several degrees of freedom (df): five to nine df for the time variable and three to five df for relative humidity. Moreover, we varied the lag period from 4 to 7 days, and we replaced minimum temperature and maximum temperature to examine the association of the cumulative temperature effect with the outcome.

We used R Version 3.6.1 and its main packages to carry out the statistical analyses for model selection, and we used the dlnm package for DLNM. R itself and all the packages used are available from the Comprehensive R Archive Network.

## Results

The summary statistics (mean, maximum, minimum, standard deviation (SD)) of the drug overdose cases and weather variables (temperature and relative humidity) are presented in Table 1. The total number of drug overdose cases recorded in Tokyo (2010–2014) was 12,937, 72.3% reside in 23 special wards in Tokyo. Mean (SD) of daily drug overdose cases was 7.1 (3.0) of which 73.9% were female. Patients aged 15–34 years accounted for half of the total cases (50.8%) followed by 35–64 years (43.3%). The daily mean temperature was 16.7 ± 8.3 °C, ranging from 0.3 °C to 33.2 °C.

**Table 1:** Summary statistics of daily drug overdose cases and weather variables in Tokyo (2010–2014)

**Variables**	**Number of cases (n)**	**Minimum**	**Maximum**	**Mean**	**SD**
**Total**	12,937	1	19	7.1	3.0
**Sex**					
Female	9570 (73.9%)	0	16	5.2	2.5
Male	3367 (26.1%)	0	8	1.8	1.4
**Age**					
15–34 years	6572 (50.8%)	0	12	3.6	2.0
35–64 years	5594 (43.2%)	0	13	3.0	1.8
≥65 years	771 (6.0%)	0	4	0.4	0.6
**Location**					
Urban (Special 23 wards)	9350 (72.3%)	0	17	5.1	2.5
Sub-urban	3587 (27.7%)	0	8	1.9	1.5
**Temperature (°C)**					
Maximum		3.7	38.3	20.5	8.4
Mean		0.3	33.2	16.7	8.3
Minimum		−1.4	30.4	13.3	8.5
**RH (%)**		19.0	95.0	60.7	15.5

SD, Standard deviation; RH, Relative Humidity

**Fig. 1:**
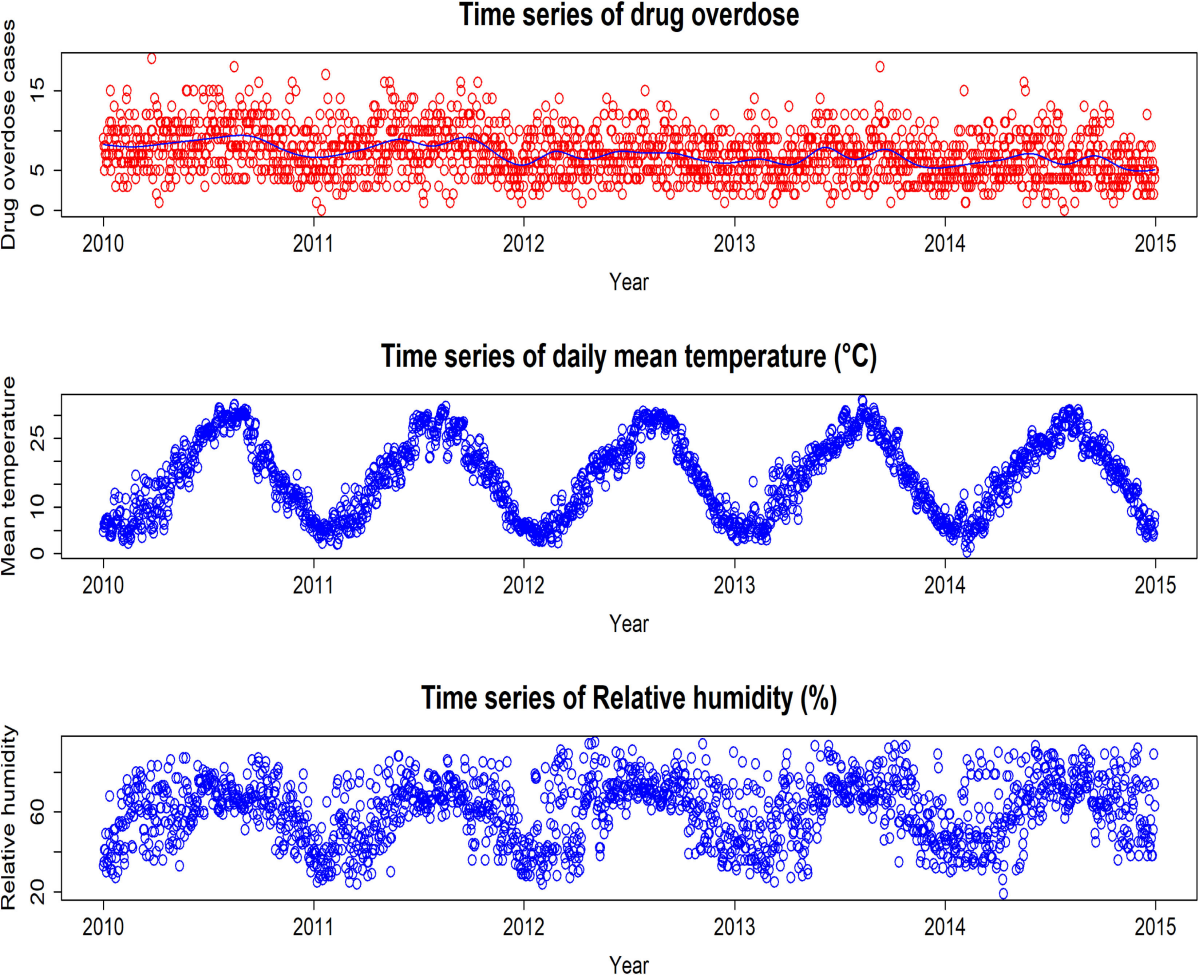
Daily time series of drug overdose cases and weather factors in Tokyo The blue line on the top figure denotes natural cubic spline function of time w/ df = 8*5 which capture seasonal patterns and time trend smoothly.

Fig. 1 displays that total overdose cases and mean temperature showed a distinct seasonality. We found that the overdose incidence occurred at a high mean temperature; however, the number decreased each year slowly.

**Fig. 2:**
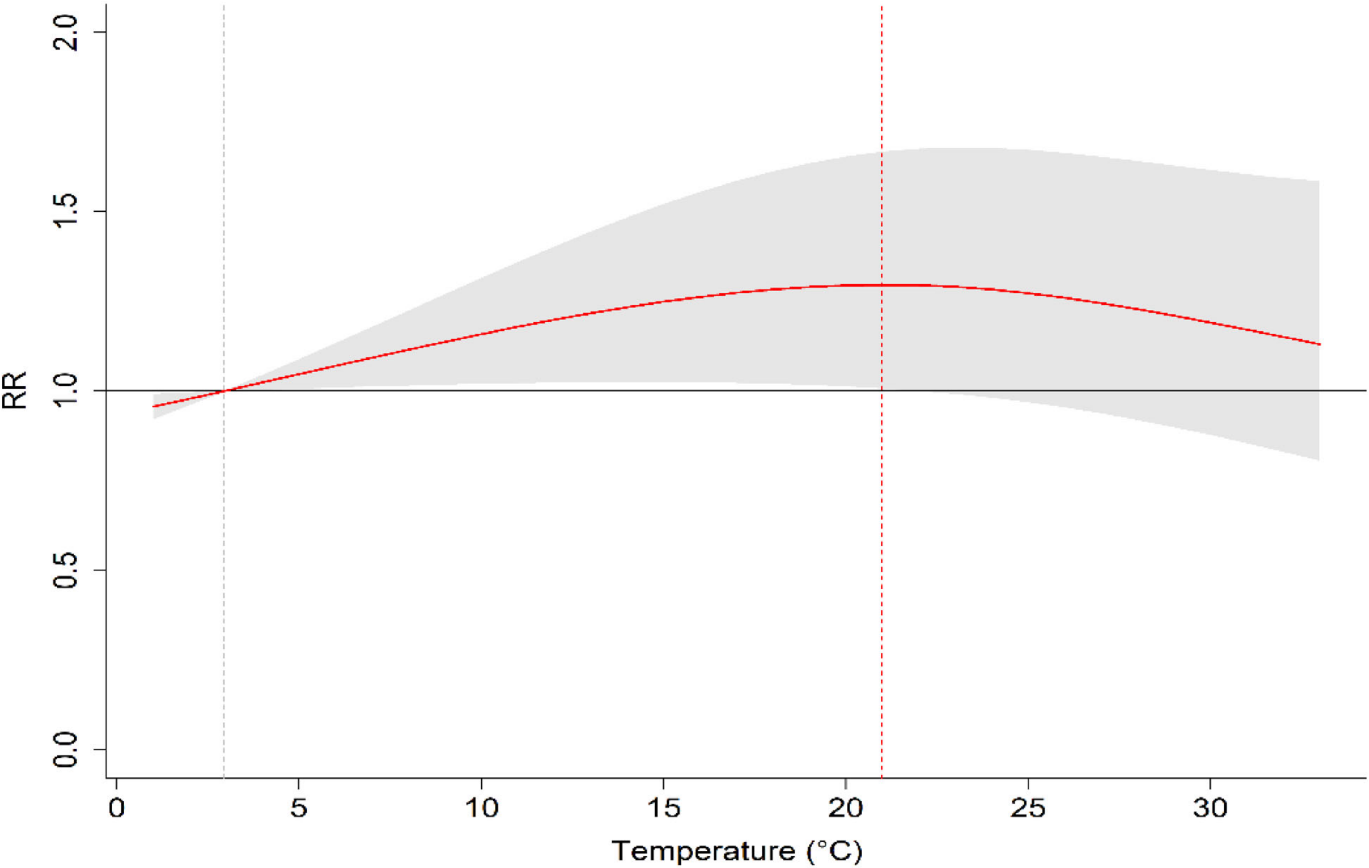
Overall cumulative association for temperature-drug overdose cases across lag 0–4 days *Note: cumulative relative risk estimates at 21 °C versus the reference temperature of 2.9 °C (95% CI shown in gray). The red dotted vertical line stands for the maximum cumulative effect estimate at the exposure.

The overall cumulative RRs of mean temperature on the number of drug overdose cases across the lag days demonstrated that higher temperature was associated with an increased risk of overdose cases, compared with the reference of 2.9 °C (Fig. 2). It shows that the maximum risk of overdose occurred at the 65^th^ percentile of the temperature distribution, which corresponds to 21 °C. The RR for the maximum overdose temperature versus the reference temperature calculated from the non-linear association was 1.30 (95% CI: 1.1–1.6) over the lag of 0–4 days; thus, the risk of overdose was 30% higher at 21 °C than at the reference temperature of 2.9 °C.

**Fig. 3:**
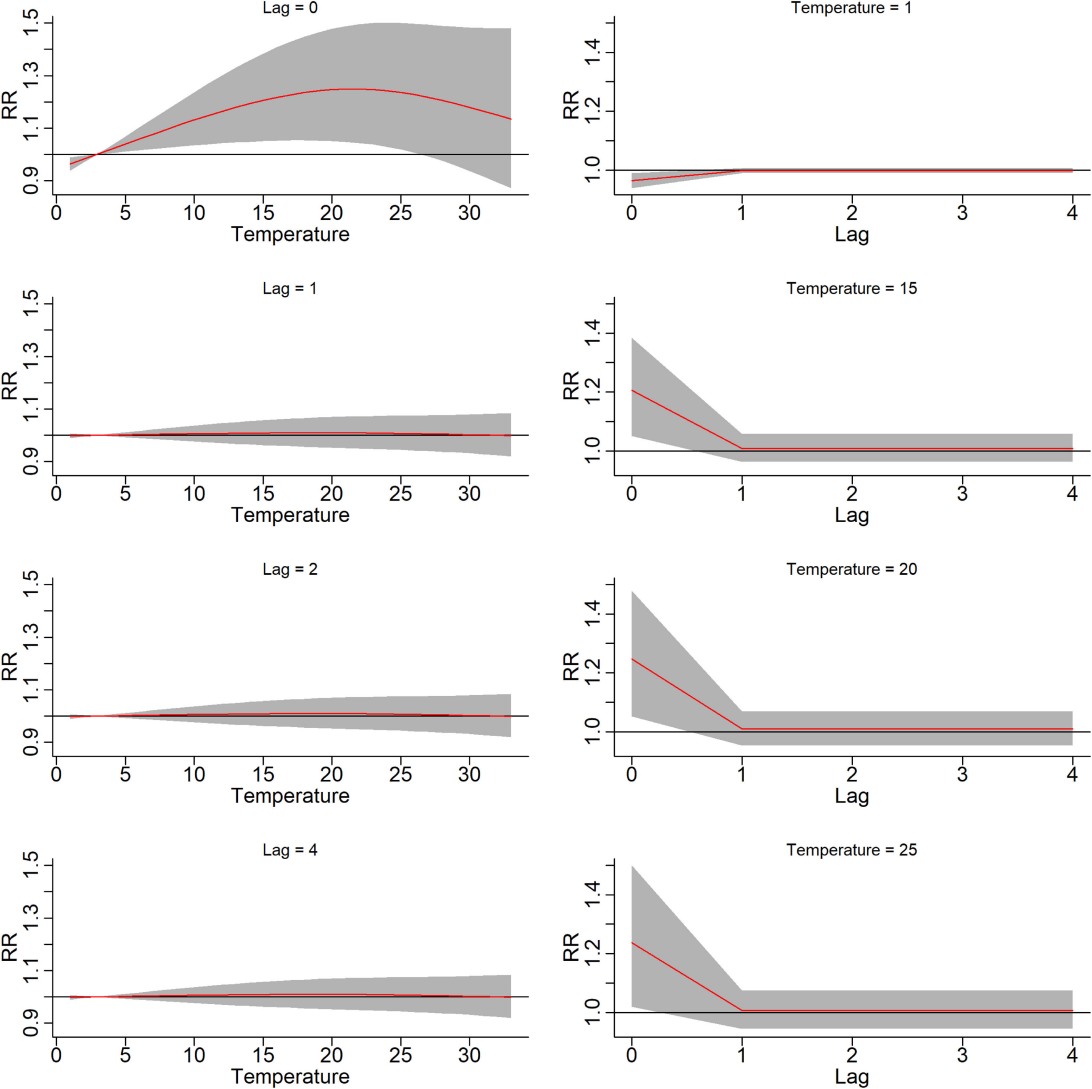
RR of temperature-specific at different lags (Left panel) and lag-specific at different temperatures (right panel) *RR, Relative risk. *(95% CIs shown in gray)

The temperature–drug overdose cases association curve varied depending on lag days (left panel, Fig. 3). The drug overdose risk increased as the mean temperature increased, mainly on current day of the event that is lag 0 day. The risk of overdose at high temperatures (above 15 °C) was relatively high on current day, and the RR began to decline when the temperature rose above 21 °C. However, at 1, 2, and 4 lag days, the effects were somewhat similar, with almost no association detected. Thus, as the left panel of Fig. 3 illustrates, the RR gradually increased with increasing temperature on the day of the event, but this relationship disappeared after the event day.

The right panel of Fig. 3 illustrates the lag–response curves at different temperatures. The RRs at high temperatures were significantly higher on the current day than on the other lag days examined. For the temperature of 20 °C, the RR was 1.30 (95% CI: 1.04–1.64) at 0 lag day, compared with the reference temperature of 2.9 °C. From 1 lag day to 4 lag days, the risk dropped and the association became almost disappearing. However, for 1 °C (top of the right panel, Fig. 3), there was a negative association with drug overdose cases, compared with the reference temperature of 2.9 °C.

The subgroup plots showed a non-linear relationship between temperature and drug overdose cases by both sexes, across age and location categories (Fig. S1, Fig. S2 and Table 2). At 21 °C, the cumulative effect estimates of self-harm by drug overdose were 1.36 (95% CI: 1.07–1.81) for female patients and 1.07 (95% CI: 0.66–1.75) for male patients, compared with the reference temperature of 2.9 °C. Across the age subgroup, we found that older adults were the most sensitive to temperature changes (Fig. S1, S2 and Table 2); the cumulative RR was 2.54 (95% CI: 0.94–6.90) for older adults, followed by 1.25 (95% CI: 0.89–1.77) for adolescents and young adults and then 1.15 (95% CI: 0.78–1.68) for middle-aged adults, compared with the reference temperature of 2.9 °C, while the 95% confidence intervals were substantially overlapped. For the location subgroups, the RR curve was higher yet insignificant for urban than for sub-urban area, with the RR of 1.28 (95%CI: 0.95–1.73) for urban area and 1.19 (95%CI: 0.75–1.88) for sub-urban area (Fig. S1 and Table 2).

**Table 2:** Cumulative relative risk of drug overdose effects across lag 0–4 days by different groups

	**Cumulative relative risk (95% CI)**
**All**	1.30 (1.10–1.67)
**Sex**	
Female	1.36 (1.02–1.81)
Male	1.07 (0.66–1.75)
**Age**	
15–34 years	1.25 (0.89–1.77)
35–64 years	1.15 (0.78–1.68)
≥65years	2.54 (0.94–6.90)
**Location**	
Urban (23 special wards)	1.28 (0.95–1.73)
Sub-urban	1.19 (0.75–1.88)

*Note: Daily mean temperature of 21 °C versus the first percentile of daily mean temperature (2.9 °C)

In the sensitivity analyses, the results of the DLNM are relatively stable when changing the degree of freedom of the time variables and other meteorological variables (Table S2). Incorporating the changes of temperature from daily maximum and minimum into the model, the lag days from 4–7 days and changing df for relative humidity, time trend respectively, the results are still relatively stable and basically show no significant change (Table S2).

## Discussion

This study aimed to ascertain the association between temperature and self-harm attempts due to drug overdose, using ambulance dispatch data from Tokyo fire stations. Our study location is Tokyo, Japan, in which the suicide rate is one of the highest among developed nations. This study depicts a non-linear association (an inverted J shape) with increasing daily mean temperature with drug overdose cases, consistent with the previous studies [18, 19]. Our study showed a relative risk of drug overdose increases with higher daily mean temperature but leveled off after 21 °C; the overall cumulative relative risk was increased by 30% at this mean temperature, compared with the reference temperature (2.9 °C) over 0–4 lag days. Previously, A multi-country study showed the suicide risk leveled off at extremely high temperatures in some countries (i.e., Japan, South Korea, and Taiwan) where populations are exposed to high temperatures [37]. A nationwide study in Japan also reported the suicide risk increased with temperature, and then after reaching the threshold, the risk leveled off; the maximum suicide temperature was estimated as 24.4 °C, and the RR was 1.26 (95% CI: 1.22–1.29) [19].

Previously, many studies have discussed the potential mechanism explaining the temperature–suicide association [10, 38] but the exact mechanism remains poorly understood. The role of environmental effects on brain function was suggested as an associative factor in suicidal behavior [10]. Increasing ambient temperature reduces emotional well-being and negatively affects mental health [16, 21, 39]. A possible biological explanation mentions that the ambient temperature influences changes in serotonin secretion, which may impact impulsiveness and aggression and possibly lead to suicidal behaviors [40–42].

Multiple studies have earlier suggested that suicide is more prevalent among men than among women [18, 34, 39, 43] but non-fatal self-harm attempt rates are higher among women than among men [40, 41, 44–46]. A possible reason for the sex difference in temperature effects is women are more sensitive to thermal changes compared with men and therefore, they need more adaptive control [21]. Regarding different age groups, those aged ≥65 years tended to show higher temperature sensitivity than in other age groups; the pattern of age group distribution is similar to previous East Asian findings where suicide risk is stronger in older adults ≥65 years [18]. The reactivation of brown adipose tissue in the older population might reduce heat tolerance and increase anxiety and agitation, thus negatively influencing mood, which might increase suicidal behavior [47–49]. As temperature changes in the environment influence brown adipose tissue, it becomes overactive, potentially increases the risk of suicidal behavior. We could not observe significant risk difference in urban vs. sub-urban area in Tokyo. Though previous nationwide study has shown variation in suicide counts between urban and rural population and has suggested larger suicidal pattern in rural areas compared to urban with possible explanation of developed public health infrastructure and more prevention program in urban than rural areas [19].

This is the first study to examine the association between ambient temperature and self-harm attempts by drug overdose in Japan. Several authors have examined the relationships between completed suicide cases and many weather factors [16, 37]; however, there has been less emphasis on the relationships between climate factors and non-fatal attempts (e.g., self-administered drug overdose). Because previous explorations of this association were relatively scarce, our study attempted to fill the void. Moreover, we also investigated the association among different age and sex groups. Hence, this study may enable predictions of possible occurrences of suicidal behavior among groups that are vulnerable to rising temperatures. Therefore, the study has the potential to aid in the initiation of attempts to prevent harmful temperature effects. The study also has implications related to the methods used to analyze the EAD’s data in the context of outdoor temperatures.

Our study has several limitations that should be considered. First, although Japan encompasses various climatic zones, our study took place in a single climate zone. The lack of large sample size and geographical variation may limit the findings and fail to show any urban-rural difference. Second, there might have been misclassification of the drug overdose diagnosis from the EAD data if final diagnosis of the patient is not collected, although most of the cases were transported to the hospitals. Moreover, the data has no detailed personal information about previous medical and drug history which might affect this suicidal behavior. Third, previous studies reported particulate matter positively associated with suicide rates, but we did not incorporate these factors in our study [50]. Finally, we monitored only one station that measured the outdoor temperature, but people might prefer to stay home for protection from extreme weather, and indoor temperatures could not be monitored using the available data.

## Conclusions

This study has suggested that elevated temperature may increase the risk of a self-administered drug overdose. We have found that females are particularly vulnerable to temperature and that those aged ≥65 years are at the highest risk among all age groups, although the subgroup analyses are inconclusive and not significant. The findings of this study support the necessity of research on the potentially harmful effects of weather factors on suicidal behavior. Our results strongly suggest the need to research the possible other illnesses to help policymakers prevent potential catastrophes. These findings may help develop a new intervention program to offer useful information to facilitate the local governments’ efforts to protect at-risk groups and establish preventive policies.

## Abbreviations

CI: Confidence interval
df: Degree of freedom
DLNM: Distributed lag non-linear model
EAD: Emergency ambulance dispatch
EMS: Emergency medical service
ICD-10: International classification of Disease Manual, 10th Version
NCS: Natural cubic spline
OD: Drug overdose
RR: Relative risk
SD: Standard deviation

## Supplementary information

**Additional file 1:** **Table S1:** Selection criteria of drugs for suicidal cases. **Table S2:** Sensitivity analyses by changing lag days and df for covariates and temperature variables. **Fig S1.** Overall cumulative association for temperature-drug overdose cases across lag 0–4 days (Location and Sex subgroups). **Fig. S2:** Overall cumulative association for temperature-drug overdose cases across lag 0–4 days by age subgroup.
