# Supplementary material for: Association between daily ambient temperature and drug overdose in Tokyo: a time-series study
Source: Environ Health Prev Med. 2022 Sep 28;27:36. doi: 10.1265/ehpm.21-00044 (PMC9556974; doi:10.1265/ehpm.21-00044)
Supplement: Supplementary file 1 — Additional file 1: Table S1: Selection criteria of drugs for suicidal cases. Table S2: Sensitivity analyses by changing lag days and df for covariates and temperature variables. Fig S1. Overall cumulative association for temperature-drug overdose cases across lag 0–4 days (Location and Sex subgroups). Fig. S2: Overall cumulative association for temperature-drug overdose cases across lag 0–4 days by age subgroup. [file ehpm-27-036-s001.docx]

**Association between Daily Ambient Temperature and Drug Overdose in Tokyo: A Time-Series Study**

Ananya Roy^1^, Md Ashraful Alam^1^, Yoonhee Kim^2^, Masahiro Hashizume^1,*^

^1^Department of Global Health Policy, Graduate School of Medicine, The University of Tokyo, Tokyo, Japan 113-0033

^2^Department of Global Environmental Health, Graduate School of Medicine, The University of Tokyo, Tokyo, Japan 113-0033

^*^Corresponding author

Masahiro Hashizume

Department of Global Health Policy

Graduate School of Medicine

The University of Tokyo

7-3-1 Hongo, Bunkyo-ku, Tokyo 1134-0033, Japan

Email: hashizume@m.u-tokyo.ac.jp

**Additional File 1**

**Supplementary information**

**Table S1:** Selection criteria of drugs for suicidal cases

| **Inclusion Criteria** | **Exclusion criteria** |
| --- | --- |
| - Drug overdose (薬物過量中毒, オーバードーズ疑い etc.) | - All the cardiac medicine used in arrhythmia, heart failure (ジギタリス, β－Blocker, テオフィリン etc.) |
| - Acute drug poisoning (急性薬物中毒) | - Illegal drugs (麻薬, 大麻, Hallucinogen, cocaine) |
| - High dose of drug (多量薬物摂取) | - ハーブ, 覚せい剤、麻酔薬 |
| - Intentional drug overdose (自殺企図による薬物服用, 自傷・薬物過量服用 etc.) | - Any unintentional or accidental poisoning (誤飲) |
| - Sedative/Hypnotic/Anti-epileptic - Non-Opioid Analgesic |  |
| - Psychotropic drug |  |
| - Mood stabilizer |  |
| - Anti- depressant |  |
| - Cold medicine |  |

**Table S2:** Sensitivity analyses by changing lag days and df for covariates and temperature variables

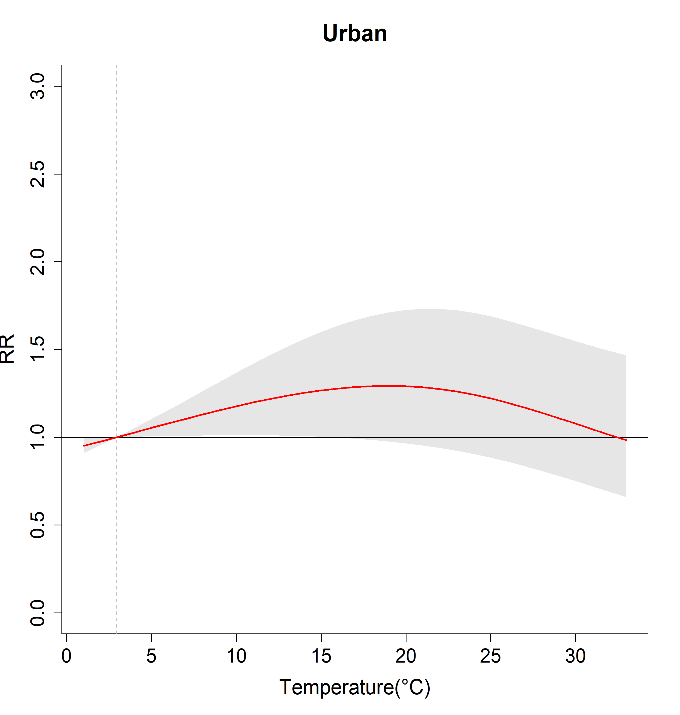

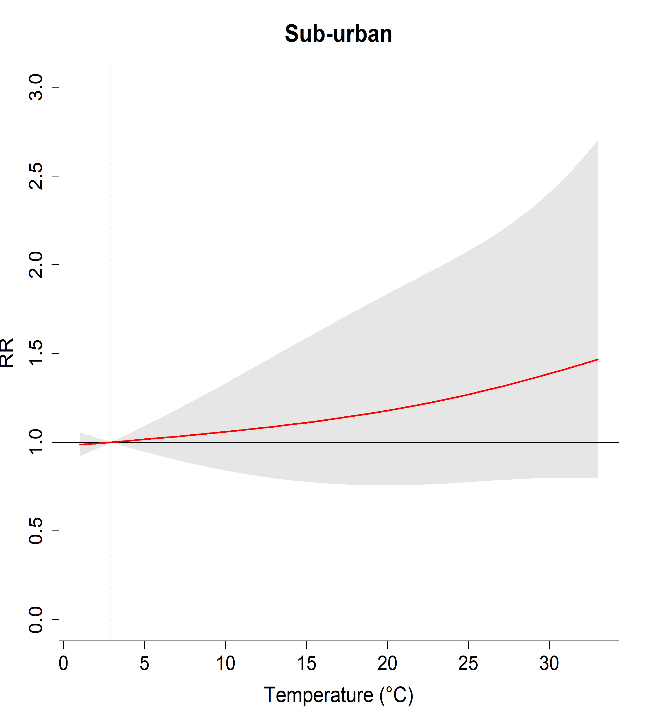

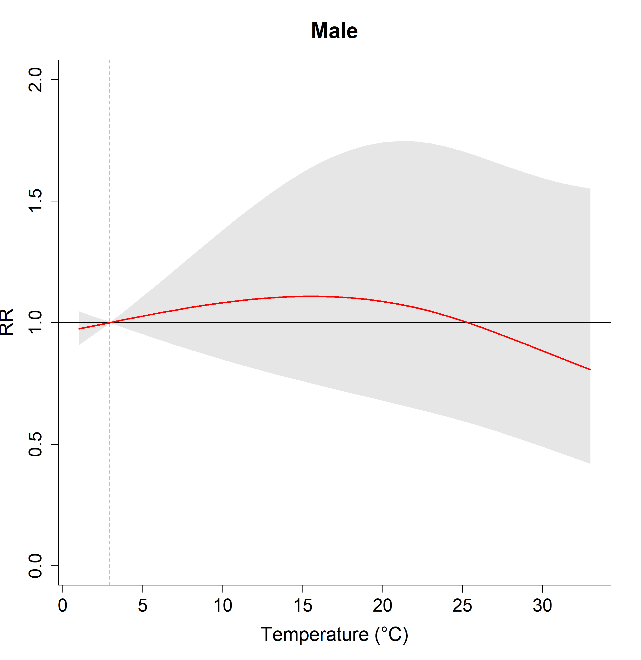

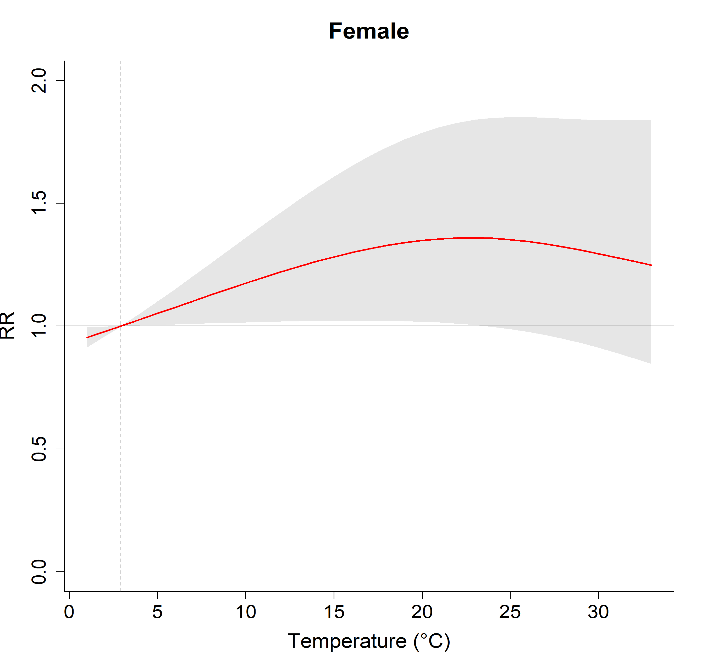


**Fig S1**. Overall cumulative association for temperature-drug overdose cases across lag 0-4 days (Location and Sex subgroups)

*Note: cumulative relative risk estimates at 21 °C versus the reference temperature of 2.9 °C (95% CI shown in gray)


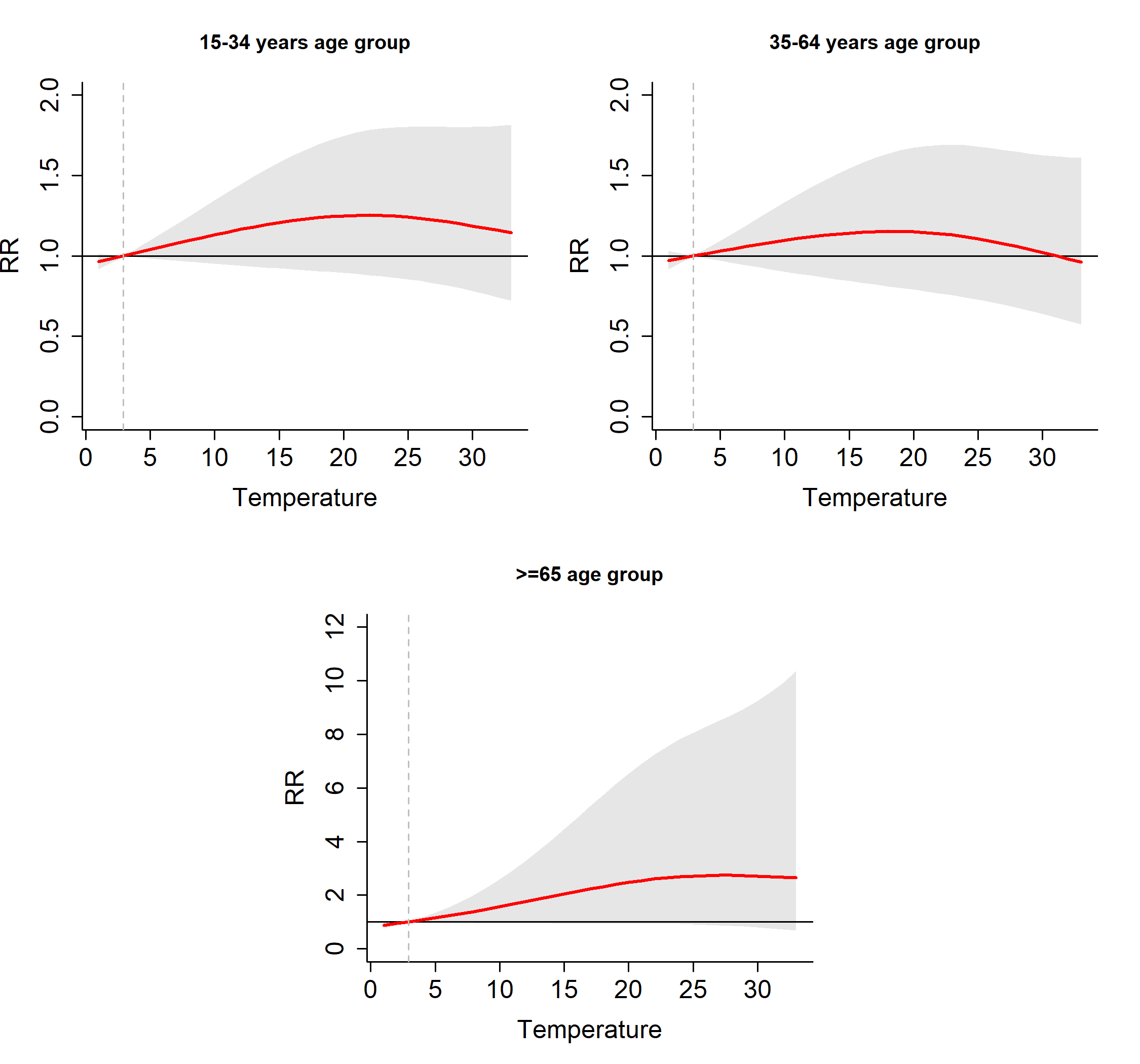


**Fig.S2**: Overall cumulative association for temperature-drug overdose cases across lag 0-4 days by age subgroup

*Note: cumulative relative risk estimates at 21 °C versus the reference temperature of 2.9 °C (95% CI shown in gray)
